# Risk factors associated with prolonged intensive care unit stay in post coronary artery bypass grafting patients with chronic kidney disease

**DOI:** 10.12669/pjms.39.2.6735

**Published:** 2023

**Authors:** Syeda Huma Zartash, Sidra Saleem, Zain Rasool, Abeera Mansur

**Affiliations:** 1 Syeda Huma Zartash (MBBS, MRCP), Director Coronary Care Unit, Doctors Hospital & Medical Center, Lahore Pakistan; 2 Sidra Saleem (M.B.B.S, FCPS, MRCP), Research Assistant Coronary Care Unit and Nephrology Department. Doctors Hospital & Medical Center, Lahore Pakistan; 3 Zain Rasool (M.B,B.S), Research Assistant Coronary Care Unit and Nephrology Department. Doctors Hospital & Medical Center, Lahore Pakistan; 4 Abeera Mansur (M.B,B.S, M.D, FACP, FASN), Consultant Nephrologist, Nephrology, Doctors Hospital & Medical Center, Lahore Pakistan

**Keywords:** Prolonged intensive care unit stay, Coronary artery bypass graft, Cardiac surgery, post op arrhythmias, Chronic kidney disease

## Abstract

**Objective:**

Prolonged intensive care unit stay not only increases hospital cost but it also prevents hospital equipment to be used by other patients who need them. The aim of this study was to identify factors that affect the duration of intensive care unit stay in post coronary artery bypass grafting patients with chronic kidney disease.

**Method:**

This is a single centered observational prospective study done on 191 post coronary artery bypass grafting patients from June 2018 to April 2019 at Cardiac Surgery Unit of Doctor’s hospital and medical center, Lahore, Pakistan. Patients above 18 years with and without chronic kidney disease were included.

**Results:**

Mean age of the patients was 57.83 years (± 9.862 SD. Logistic regression analysis shows that patients with post op arrhythmias had the strongest positive association with prolonged intensive care unit stay (OR:11; p value :<0.01), followed by recent myocardial infarction less than 90 days pre coronary artery bypass grafting (OR:5.93; p value:<0.01), shock (OR:3.93;p value:0.04) and acute kidney injury (OR :2.08;p value:0.04). 37.5% chronic kidney disease patients with recent myocardial infarction less than 90 days pre coronary artery bypass grafting and 51.4% patients of chronic kidney disease found with acute kidney injury, showed significant association with p values less than 0.05.

**Conclusion:**

Post op arrhythmias, recent myocardial infarction, shock and acute kidney injury are independent risk factors causing prolonged intensive care unit stay in post coronary artery bypass grafting patients.

## INTRODUCTION

Intensive Care Unit (ICU) stay is associated with increased cost and limited resources in developing countries. Prolonged ICU stay not only increases hospital cost but it also prevents hospital equipment to be used by other patients who need them. As the guidelines (American college of cardiology/American Heart Association 2011) recommend coronary artery bypass grafting (CABG) over percutaneous coronary intervention (PCI) for patients with diabetes mellitus (DM), multi-vessel coronary artery disease and with low ejection fraction (EF); an increased incidence of CABG has been observed over the last decade. This has posed an extra burden on availability of ICU equipment especially in developing countries where limited resources are a matter of real concern. Prolonged ICU stay due to various complications increases a patient’s cost, as well as limits the availability of ICU equipment to other patients who need it.^1^ This puts an additional burden on health care facilities in developing countries.

It has been reported that approximately 19% - 45% of the patients may need prolonged intensive care after open heart surgery.^1^-^6^ In some studies, advanced age, female gender, reduced left ventricular function, arrhythmia, inotropic agent support and intra-aortic balloon pump (IABP) requirements have been identified as risk factors for prolonged intensive care.^2^-^6^ However, the impact of chronic kidney disease (CKD) on prolonged ICU stay in post CABG patients has not been well studied. The aim of this study was to identify risk factors causing prolonged ICU stay in post CABG CKD and non CKD patients that can help us in timely management and resource planning. The increasing prevalence of CKD patients in our region was the key driving force to include these patients in our study .According to high quality studies, highest CKD prevalence in Pakistan was reported as 29.9% and the lowest prevalence was 12.5%.^7^

## METHODS

This is a single centered observational prospective study done on 191 post CABG patients at Cardiac Surgery Unit of Doctor’s Hospital and Medical Center, Lahore, Pakistan, from June 2018 to April 2019. Study was approved by the ethics committee and Institutional review board of the Doctor’s Hospital and Medical Center with IRB approval number: IRB/06/2018/01 dated 09/06/2018. CABG patients, above 18 years (Mean age was 57.83 years ± 9.862 SD), with and without CKD and those who had coronary angioplasties in the past were included. However, patients with redo CABG and valve surgery were excluded.

ICU length of stay (LOS) was categorized as normal (less than 48 hours) and prolonged (more than 48 hours). CKD and AKI were classified according to the KDIGO guidelines. Patients were categorized as double vessel and triple vessel coronary artery disease. Grafts were classified as arterial or venous or both. Left ventricle (LV) dysfunction was graded based on ejection fraction (EF) according to the New European Society of Cardiology guidelines that suggest that patients with heart failure (HF) should be categorized as HFpEF (heart failure with preserved EF ≥50%), HFrEF (heart failure with reduced EF <40%), and HFmrEF (HF with midrange EF 40%-49%).

Diastolic dysfunction was defined according to 2016 DIASTOLOGY GUIDELINES, using the echocardiographic parameters of LV diastolic dysfunction including e’, E/e’, left atrial volume index and peak TR velocity. Arrhythmias included SVT (Supraventricular tachycardia), atrial fibrillation and VT (ventricular tachycardia). In our study cohort, shock was considered as present in those patients who required use of inotropic support /devices to maintain systolic B.P at or above 90mmHg.Duration of bypass is the time from commencement of surgery to the end of cardiopulmonary bypass. Data analysis was done using SPSS version 25. The effect of different patient variables and perioperative risk factors on prolonged ICU stay were analyzed using Pearson Chi square test and logistic regression analysis.

## RESULTS

**Fig.1:**
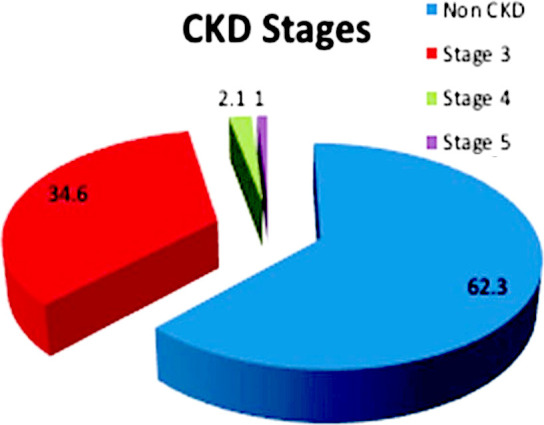
Patients according to CKD Stages.

Mean age was 57.83 years (± 9.862 SD) .32 (16.8%) were females and 159 (83.2%) were males.145 patients (75.9%) were hypertensive and 110 (57.6%) were diabetics and all had dyslipidemias (Table-I). 18/191(9.4%) had prior H/O PCI. 4/191 patients who had CABG had double vessel disease. Rest had triple vessel disease. 170/191 (89%) had obstructive right coronary artery, 177/191 (92.7%) had obstructive left circumflex, 186/191 (97.4%) had obstructive left anterior descending and 54/191 (28.3%) had left main stem disease. Obstructive lesions being labeled as more than 80% stenosis. 190/191(99.5%) patients had venous grafts. And 135/191(70.7%) had arterial grafts. 66 patients had CKD stage three, four had CKD stage four and two had CKD stage five as shown in (Fig.1).

**Table-I:** Demographics.

Age		57.83 ± 9.862 years
Gender		159(82%) Male, 32(16.8%) Female
BMI (kg/m2)		28.28 ± 4.66
DM		110(57.6%)
HTN		145(75.9%)
Dyslipidemia		191(100%)
Smoking		36(18.8%)
CKD stage	Non CKD	119(62.3%)
	Stage 3	66(34.6%)
	Stage 4	4(2.1%)
	Stage 5	2(1.0%)
AKI		44(23%)

Results of logistic regression analysis (Table-II) shows that patients with post op arrhythmias had the strongest positive association with prolonged ICU stay (OR:11; p value :<0.01), followed by recent MI less than 90 days pre CABG (OR:5.93; p value:<0.01), shock (OR:3.93;p value:0.04) and AKI (OR :2.08;p value:0.04).CKD did give some association with prolonged ICU stay, OR of 1.35, but p value was not significant. Patients with DM were 0.38 times significantly less likely to be found with prolonged ICU stay. LVEF <35% did not give any significant positive association with prolonged ICU stay.

**Table-II:** Association of Risk Factors with Prolonged ICU Stay using Binary Logistic Regression.

FACTORS	Number of patients with prolonged ICU stay (N=56)	OR (95% C.I)	P-VALUE
DM	23 (41%)	0.38(0.20,0.72)	0.003*
SHOCK	6 (10.7%)	3.93 (1.06, 14.54)	0.04*
ARRHYTHMIA (POST-CABG)	29 (51.8%)	11.0 (4.9,24.2)	<0.01*
AKI	19 (40%)	2.08 (1.02,4.23)	0.04*
CKD	24 (42.8%)	1.35 (0.72, 2.56)	0.34
LVEF <35%	11 (19.6%)	0.5 (0.23, 1.07)	0.07
Recent MI (LESS THAN 90 DAYS PRE-CABG)	11 (19.6%)	5.93 (2.0,17.4)	<0.01*

**Dependent variable :** Prolonged ICU Stay

**Independent variables** : DM, Shock, Arrhythmia, AKI, CKD, Dialysis, LVEF <35%

* odds ratio obtained using Binary logistic Regression considered significant with p<0.05

**Table-III:** Risk Factors causing prolonged ICU Stay in CKD and non CKD patients.

Factors		CKD				p-value

		NONE		YES		
	
		n	%	n	%	
DM	None	56	47.1	25	34.7	0.09
	Yes	63	52.9	47	65.3	
Shock	Not present	112	94.1	69	95.8	0.60
	Present	7	5.9	3	4.2	
Arrhythmia post. CABG	Negative	95	79.8	55	76.4	0.57
	Yes	24	20.2	17	23.6	
MI	Negative	91	76.5	45	62.5	0.03*
	Present	28	23.5	27	37.5	
EF (%)	<35	10	8.4	8	11.1	0.55
	35-55	52	43.7	35	48.6	
	>55	57	47.9	29	40.3	
Diastolic Dysfunction	None	45	37.8	25	34.7	0.97
	Grade 1	39	32.8	25	34.7	
	Grade 2	29	24.4	18	25.0	
	Grade 3	6	5.0	4	5.6	
AKI	No	113	95.0	35	48.6	<0.01*
	AKI	6	5.0	37	51.4	

Further analysis revealed a strong correlation of on pump surgeries with prolonged ICU stay as (p value: 0.04) and 50% of the CKD patients on pump had prolonged ICU stay. The percentage of patients with prolonged ICU in on pump surgeries (66.7%) was found to be much higher than in off pump surgeries (28.1%). However, it was not statistically significant due to the small number of on-pump surgeries done in our hospital.

## DISCUSSION

Risk factors causing prolonged ICU stay in post CABG patients with CKD are not well studied especially in our south Asian population. Thus, this study will serve as a reference for the future and would help in the development of guidelines to reduce prolonged ICU stays in an already overloaded healthcare system. We studied 191 patients with mean age of 57.83 years and a female to male ratio of (1:5).Risk factor prevalence in our study showed 57.6%diabetics,75.9% hypertensive and 100% dyslipidemics which is comparable to another local study demographics where mean age was 58 years, diabetics were 42.7%,hypertensives were 64.9% and dyslipidemics were 71.8%.^8^ There is no consensus on the definition of prolonged ICU LOS. Prolonged ICU stay is accepted from 48 hours to 10 days which is considered as a wide range.

We considered a duration of more than 48 hours as a prolonged ICU stay. Heimrath et al^3^ also took prolonged ICU stay as more than 48 hours whereas Brucerius^2^ and Elayne Kelen de^9^ considered prolonged ICU stay as more than 72 hours. 56/191 (29.3%) patients enrolled in our study had prolonged ICU stay which closely matches the frequency of patients with prolonged ICU stay (34.2%) after CABG in a tertiary care center for cardiovascular patients, Tehran, Iran.^10^ While the patient’s age was not found to be associated with prolonged ICU stay, amongst the pre op characteristics, recent MI less than 90 days pre CABG had the strongest association. It is observed that patients with recent MI take more time to achieve hemodynamic stability and also are more prone to arrhythmias. Messaoudi et al^11^ also showed recent MI as one of the most important factors that prolonged ICU stay.

Heimrath et al^3^ observed that 598/3139 (19%) of the patients undergoing CABG had prolonged ICU stay of more than 48 hours. This study reported patients with advanced age, female gender, recent MI, unstable angina, DM and patients with emergency surgeries to be associated with prolonged ICU stay. However, our study showed a negative association with diabetes mellitus and no association of age, gender or emergency surgery with prolonged ICU stay. Post op arrhythmias showed a strong association with prolonged ICU stay in our study group, however, in another study, patients with atrial fibrillation preoperatively were associated with an almost 6.3 times greater probability of having an ICU stay of more than two days.^12^

A study by Valderrabano^13^ gives a strong association of post op supraventricular arrhythmia with prolonged ICU stay and post op ventricular arrhythmia with increased hospital mortality. Abrahmyan^14^ shows arrhythmia as the most frequent postoperative complication (17.6%) and significantly associated with prolonged ICU stay. Atrial fibrillation precipitates ventricular dysfunction thus causing hemodynamic instability. Hemodynamic instability causes shock which leads to AKI. All of these together lead to a vicious circle aggravating fluid overload thus causing prolonged intubation and prolonged ICU stay.^15^ In our study, 72/191 (37.6%) patients had CKD three to five. CKD did give a positive association with prolonged ICU stay, however, AKI was more strongly associated with prolonged ICU stay with an OR of 2.08(p value :0.04) similar to a study conducted on 513 patients by Tunc M et al^16^ which showed post op arrhythmias and renal dysfunction as factors prolonging ICU stay.

Association of AKI with mortality, morbidity, CKD, and prolonged hospitalization especially in critically ill patients and patients admitted to ICU is well documented in the literature, which reflects the worldwide importance of early detection and prevention with the aim of decreasing death and morbidity.^17^-^21^ A systemic review done by Almashrafi et al^22^ shows association of increased age, atrial fibrillation and renal dysfunction with prolonged ICU LOS.A similar study done by Atoui R^23^ on 426 patients shows significant associations of ejection fraction <40% (p=0.04), and history of renal failure (p=0.001).Whereas, a locally conducted study in our region showed some benefit of tight glycemic control(in pre and post CABG patients) on the length of ICU stay.^24^

The percentage of patients with prolonged ICU stay in on pump surgeries (66.7%) was found to be much higher than in off pump surgeries (28.1%). However, it was not statistically significant due to the small number of on pump surgeries done in our hospital. Similarly, Brucerius J et al^2^ determined low risk of prolonged ICU stay in off pump CABG patients as compared to on pump CABG.

### Limitation:

Most of our patients were CKD stage three, however patients with CKD stage four and five were limited. There was lack of significant data on CKD patients for comparison with our study.

## CONCLUSION

Post op arrhythmias, recent MI less than 90 days pre CABG, shock and AKI are independent risk factors causing prolonged ICU stay in post CABG patients. Among the CKD cohort, patients with recent MI less than 90 days pre CABG and those who had AKI gave the strongest association with prolonged ICU stay. Devising a simple predictive score system to assess hemodynamic instability can help timely identification and management of patients at risk of prolonged ICU stay.

### Author’s contributions:

**SHZ:** Conception and design, acquisition and interpretation of data, drafting of manuscript, revising and editing the manuscript critically for important intellectual contents, is responsible and accountable for the accuracy and integrity of the work.

**SS:** Conception and design,, drafting of manuscript, revising the manuscript.

**ZR:** Acquisition analysis and interpretation of data, revising and editing the manuscript critically.

**AM:** Conception and design, drafting of manuscripts, research coordination and management.
